# Synthesis and Properties of Semicrystalline Poly(ether nitrile ketone) Copolymers

**DOI:** 10.3390/polym16020251

**Published:** 2024-01-16

**Authors:** Jiang Zhu, Chao Mo, Lifen Tong, Xiaobo Liu

**Affiliations:** 1 Sichuan Aerospace Fenghuo Servo Control Technology Co., Ltd., Chengdu 611130, China; zjuestc434@163.com; 2 School of Materials and Energy, University of Electronic Science and Technology of China, Chengdu 611731, China; mochao202121@163.com; 3 Sichuan Province Engineering Technology Research Center of Novel CN Polymeric Materials, Chengdu 611731, China

**Keywords:** poly(ether nitrile ketone), isothermal heat treatment, mechanical properties, crystalline

## Abstract

As a high-performance engineering plastic, polyarylene ether nitrile (PEN) is widely used in many fields. The presence of cyano groups of PEN ensures its good adhesion to other substrates, but the inherent low crystallinity of PEN limits its application. In this work, the poly(aryl ether ketone) segment was introduced into PEN via copolymerization using both 2,6-Dichlorobenzonitrile and 4,4′-Difluorobenzophenone as the starting reagents to prepare poly (ether nitrile ketone) (BP-PENK). The effect of composition and thermal treatment on the crystallization behavior and properties of poly (ether nitrile ketone) were systematically studied. It was found that when the content of DFBP is 30%, the copolymer BP-PENK30 had the best mechanical properties, with a tensile strength of 109.9 MPa and an elongation at a break value of 45.2%. After thermal treatment at 280 °C for 3 h, BP-PENK30 had the highest crystallinity with a melting point of 306.71 °C, a melting enthalpy of 5.02 J/g, and crystallinity of 11.83%. Moreover, with the increase in crystallinity, the dielectric constant and energy density increased after thermal treatment. Therefore, the introduction of poly(aryl ether ketone) chain segments and thermal treatment can effectively improve the crystallization and the comprehensive properties of PEN.

## 1. Introduction

In certain harsh circumstances, high-performance engineering plastics are needed to meet the application requirement [1,2,3,4,5,6,7,8]. Polyarylene ether nitrile (PEN) is a kind of polyaryl ether polymer with the cyano group in its side group. PEN not only possesses the characteristics of high-performance engineering plastics such as high strength, high modulus, and high-temperature resistance [9,10,11] but also has excellent properties such as self-flame retardant, low water absorption, and friction resistance. In addition, PEN has a flexible molecular design, which can be applied in the field of adsorption. Because of these merits, PEN shows great potential in the fields of aerospace, the automobile industry, electronics, and other fields [12,13,14].

However, the crystallinity of PEN is generally low, and the mechanical properties and solvent resistance of amorphous PEN are slightly worse. For example, amorphous PEN such as bisphenol A-type PEN (BPA-PEN) and phenolphthalein-type PEN (PP-PEN) can be dissolved in the most common solvents (NMP, DMAc, DMF, DMSO, etc.), while semi-crystalline PENs such as hydroquinone-type PEN (HQ-PEN) and biphenylene-type PEN (BP-PEN) need to be heated in order to dissolve some highly polar solvents [15] (BPA-PEN, PP-PEN, HQ-PEN, and BP-PEN refer to polyarylene ether nitrile obtained by reacting different bisphenol monomers with DCBN monomers, whose bisphenol monomers are bisphenol A, phenolphthalein, hydroquinone, and biphenylene, respectively). In addition, the highest service temperature of amorphous polymers is their glass transition temperature, while crystalline polymers can be used above their glass transition temperature. Therefore, improving the crystallinity of PEN is one of the hot spots in PEN research. Commonly used methods to improve crystallinity include adjusting the polymer structure, increasing the external field [16], adding a nucleating agent, and so on [17,18,19,20,21,22,23]. Tu and You et al. prepared HQ/ BPA-PEN with different structures using the polycondensation reaction and investigated the effect of the temperature field on the crystallization properties of the material. Tong and Wang et al. added BPA-PEN to HQ/RS-PEN to improve the fluidity of the material and increase the crystallinity of HQ/RS-PEN. At the same time, it was verified that the crystallization rate of HQ/RS-PEN was accelerated under the shear field [24].

Poly (ether ether ketone) (PEEK) is a high-performance engineering polymer material. It has good mechanical properties [25,26,27] and thermal stability due to the presence of a polar carbonyl group and rigid benzene ring in its molecular chain [28,29,30,31,32]. The existence of ether bonds in the main chain is beneficial improving the flexibility of polymer chains and the processing properties of materials [33,34,35,36]. As a semi-crystalline material, PEEK usually has a high crystallinity between 30 and 35% [37]. Therefore, PEEK has excellent high-temperature resistance, chemical resistance, mechanical properties, self-flame retardant, and so on. However, PEEK has a high crystallinity, so it is easy to precipitate during synthesis. At the same time, the processing temperature is also particularly high, resulting in processing difficulties [38].

Poly (ether nitrile ketone) (PENK) with polyaryletherketone and polyarylethernitrile chain segments may have better processability than PEEK, and at the same time, PENK may show better thermal properties, mechanical properties, and solvent resistance than PEN [39]. Therefore, BP-PENK was prepared via the copolymerization of DCBN, DFBP, and BP in this work. The effects of varying the ratio of raw materials and high-temperature heat treatment on the crystallinity, mechanical properties, and energy storage density were investigated.

## 2. Experimental Section

### 2.1. Materials

Biphenol (BP, 99%) was purchased from Hunan Dacheng Medical Chemical Co., Ltd., Loudi, China. 2,6-Dichlorobenzonitrile (DCBN, 99%) was obtained from Lianyungang Tianchen Chemical Co., Ltd., Jiangsu, China. 4,4′-Difluorobenzophenone (DFBP, 99%) was purchased from Shanghai D&B Biotechnology Science and Technology Co., Ltd., Shanghai, China. Potassium carbonate (K_2_CO_3_, AR), ethanol (AR), N-Methyl pyrrolidone (NMP, AR), toluene, and hydrochloric acid were purchased from Chengdu Kelong Chemical Co., Ltd., Chengdu, China.

### 2.2. Synthesis of BP-PENK Copolymers

**Figure 1:**
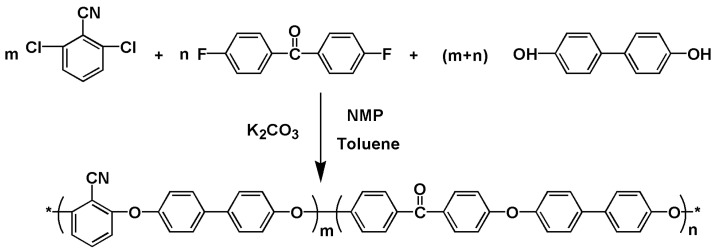
Synthetic route for BP-PENK copolymers.

BP-PENK copolymers were synthesized via the condensation polymerization of DCBN, DFBP, and BP, using K_2_CO_3_ as a catalyst in NMP and toluene. Figure 1 shows the synthetic route of BP-PENK copolymers. Table 1 shows the number of raw materials for BP-PENK copolymers. The prepared copolymer was crushed, soaked in hydrochloric acid, washed in boiling water, and dried. Finally, secondary purification was performed. The synthesis of the PENK30 polymer was used as an example. First, 0.1050 mol DCBN, 0.0450 mol DFBP, 0.1500 mol BP, 0.2100 mol K_2_CO_3_, 81 mL NMP, and 27 mL toluene were added to a three-necked flask, and stirring and heating were started. When the temperature rose to 140 °C, the phenomenon of dehydration reflux appeared in the flask, and the reaction was continued at this temperature for 2.5 h. Next, water and toluene in the water separator were slowly released so that the reaction temperature in the flask was gradually increased to 185 °C. The reaction was carried out at this temperature for about 1 h, and there was no clear change in the viscosity of the reactants. Then, the material in the three-necked flask was poured into deionized water and subsequently crushed to obtain poly(ether nitrile ketone) powder. The poly(ether nitrile ketone) powder was added to a dilute hydrochloric acid solution, which was configured to remove residual potassium carbonate. The material was subsequently boiled with water several times and filtered to obtain poly(ether nitrile ketone) powder. The obtained PENK powder was then dried in an oven at 110 °C for 12 h. Finally, PENK30 was dissolved in NMP at a solid–liquid ratio of 1:12 and subjected to secondary purification according to the above procedure.

### 2.3. Preparation of BP-PENK Films

**Figure 2:**
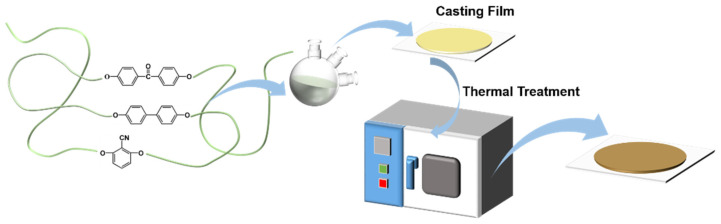
Preparation process of PENK films.

Figure 2 shows the preparation process of PENK films. BP-PENK copolymers (2 g) were added into a 100 mL three-necked flask containing 30 mL of NMP under heating and stirring. After it was completely dissolved, it was poured onto a clean glass plate and dried at 80 °C (1 h), 100 °C (1 h), 120 °C (1 h), 160 °C (1 h), 200 °C (2 h) in the oven. Finally, it was cooled to room temperature to obtain the BP-PENK film.

### 2.4. Isothermal Treatment of BP-PENK

BP-PENK films were placed in the high-temperature oven, and the temperature was set to 260 °C, 270 °C, 280 °C, and 290 °C for 3 h and cooled naturally to room temperature.

### 2.5. Characterization

The chemical structure of the copolymer was characterized using Fourier Transform Infrared Spectroscopy (FTIR, INVENNIO R). The intrinsic viscosity of the copolymer was measured using the Ubbelohde viscometer via the one-point method at a temperature of 75 °C ± 0.1 °C, and the intrinsic viscosity was obtained using Equation (1):

(1) $$\left[\eta \right]=\frac{\sqrt{2\left(\eta_{sp}{-\eta }_{r}\right)}}{c}$$

Here, (*η*) is the intrinsic viscosity, *c* is the NMP solution concentration of the prepared PEN, *η_r_* is the relative viscosity, and *η_sp_* is the increasing specific viscosity. The thermal properties of the copolymer were tested using the TA instrument DSC-Q100 and TA instrument TGA-Q50. The DSC test was heated from room temperature to 350 °C at a rate of 10 °C/min under nitrogen protection of 50 mL/min. The TGA test was heated from room temperature to 700 °C at a rate of 20 °C/min under nitrogen protection of 50 mL/min. Mechanical properties were tested on a series desktop electromechanical universal testing machine (SANS CMT6104, Shenzhen, China) in the tensile mode at a stretching rate of 5 mm/min. The dielectric properties of the materials were tested using TH2826/an LCR dielectric analyzer (Tonghui Electronic Co., Ltd., Shenzhen, China). Wide Angle X-ray Diffraction (XRD) was performed on the X-ray diffractometer (Rigaku Ultima IV, Tokyo, Japan) using a copper Kα radiation source with a speed of 8°/min at room temperature. The energy storage density was measured using the voltage breakdown tester (Beijing Air Timos Instrument Co., Ltd., Beijing, China).

## 3. Results and Discussion

### 3.1. Synthesis of BP-PENK

**Figure 3:**
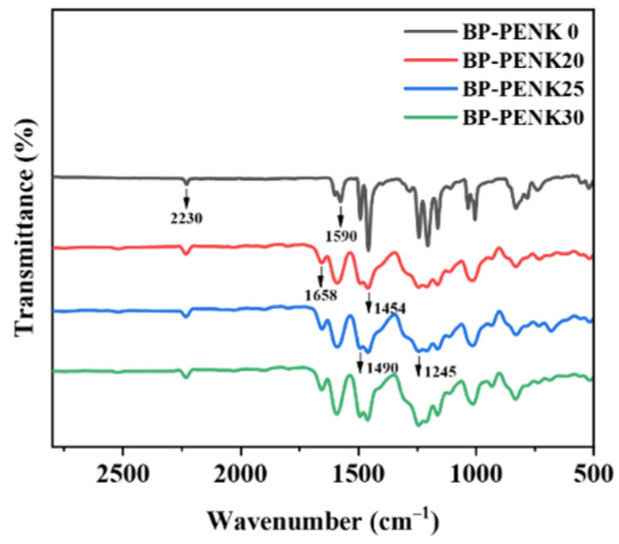
FTIR spectra of BP-PENK copolymer films.

The chemical structure of the copolymer was characterized using FTIR, and Figure 3 is the FTIR spectrum of BP-PENK. The characteristic absorption peak at 2230 cm^−1^ belongs to the pendant nitrile group (-CN). The characteristic absorption peak of the stretching vibration of the ketone carbonyl group (C=O) was observed near 1658 cm^−1^. The absorption peaks near 1590 cm^−1^, 1490 cm^−1^, and 1454 cm^−1^ belong to the characteristic absorption peaks of the benzene ring. The asymmetric characteristic absorption peak of the aromatic ether bond (-O-) is 1245 cm^−1^. The FTIR spectra of BP-PENK0 do not contain the ketocarbonyl-stretching vibration peaks in the poly(aryl ether ketone) chain segments; in addition, PENK20, PENK25 and PENK30 contain the ketone carbonyl groups. The obtained FTIR spectral results indicated the successful synthesis of the target polymers. The molecular weight of the copolymer was indirectly characterized by the intrinsic viscosity. The intrinsic viscosity of BP-PENK20, BP-PENK25 and BP-PENK30 was found to be 1.39 dL/g, 1.64 dL/g, and 1.81 dL/g, respectively. Such results suggest that more polyaryletherketone moieties are beneficial for obtaining polymers with a higher molecular weight.

### 3.2. Thermal Properties of BP-PENK Polymers

**Figure 4:**
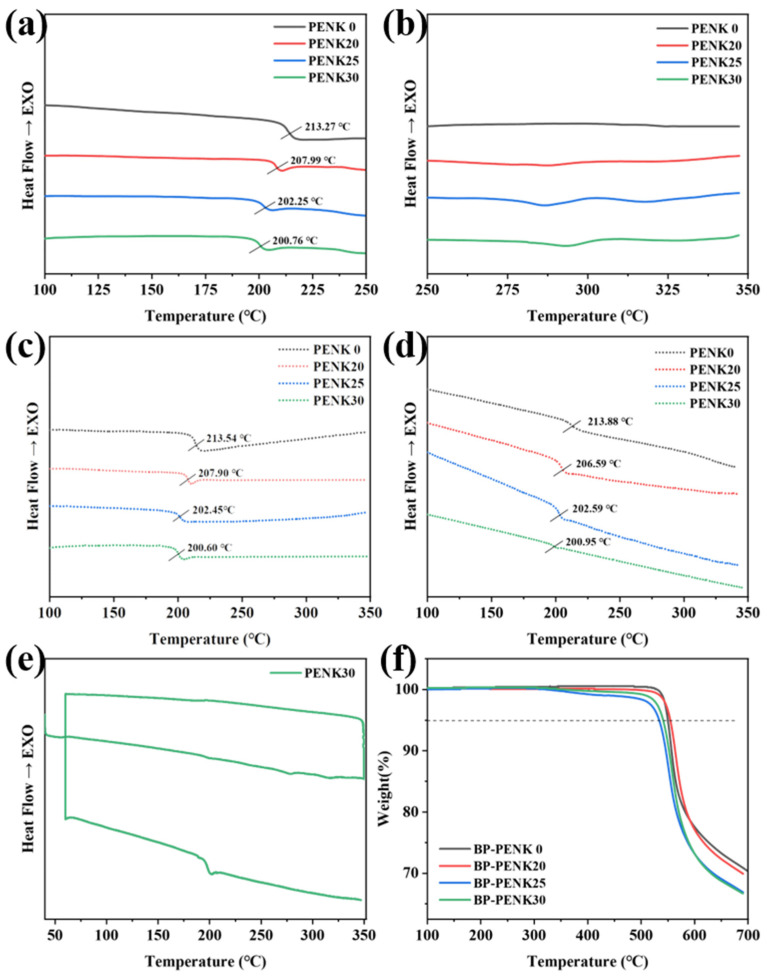
Thermal performance chart of the BP-PENK copolymer (**a**) *T_g_* curve of BP-PENK, (**b**) *T_m_* curve of BP-PENK, (**c**) DSC curve of the removal thermal history with warming at 10 °C/min rate and cooling at 10 °C/min rate, (**d**) DSC curve of thermal history removal after warming at 2 °C/min rate and cooling at 2 °C/min rate, (**e**) Cyclic DSC curve of PENK30 warmed up at a rate of 2 °C/min and cooled down at a rate of 2 °C/min followed by warming up at 10 °C/min. (**f**) TGA curve of BP-PENK.

The glass transition temperature (*T_g_*) and melting of BP-PENK were measured and analyzed using a differential scanning calorimeter (DSC). The glass transition temperatures (*T_g_*) of the polymers are shown in Figure 4a, and the detailed thermal data are shown in Table 2. The *T_g_* of the polymers are all higher than 200 °C and have a high service temperature. The *T_g_* of biphenyl-type Poly (ether ether ketone) (BP-PEEK) is lower than that of BP-PEN. Therefore, the *T_g_* of the polymers decreases gradually with the increase in the polyaryletherketone chain segments in the polyetherketonitrile, which is evidence of successful polymer synthesis. The melting of the polymer is shown in Figure 4b, and the polymer has weak crystallization. With the increase in polyaryletherketone chain segments in the polymer, the melting peaks were shown on the DSC, which indicated that the addition of polyaryletherketone was helpful for the crystallization of the polymer. When the DSC scanning reached 350 °C at 10 °C/min, and then cooled to room temperature at 10 °C/min, a second temperature scanning was performed to obtain the DSC curve of the polymer removal heat history, as shown in Figure 4c. The heating rate and cooling rate were both 2 °C/min and the DSC curve of the removal heat history of the polymer is shown in Figure 4d. The *T_g_*′ and *T_g_*″ of the polymer were almost unchanged due to the weak crystallization ability of the polymer at this condition. However, the melting peak of the polymer disappeared after the removal of the heat history; this was due to the cooling down of the temperature, which was so fast that the polymer’s internal nuclei could not be formed in time, the crystals could not grow, the polymer at this time was in the amorphous state, and there was no crystal-melt to absorb heat. This also indicates that the polymer crystallized slowly. This is evidenced by the cyclic DSC of PENK30 in Figure 4e. In addition, the thermal stability of the BP-PENK polymer was tested using thermogravimetric analysis (TGA). The TGA curve of the polymer is shown in Figure 4f. The 5% decomposition temperatures (*T_d_*_5%_) of the polymers were all higher than 530 °C, and the copolymers had good thermal stability. PENK20, PENK25, and PENK30 underwent the first thermal weight loss at around 300 °C, which might be due to the residual solvent NMP within the film, indicating the easier removal of the residual NMP solvent from the PENK0 structure [40].

### 3.3. Mechanical Properties of BP-PENK Polymers

Mechanical properties are an important index of polymer structural materials. Specific data on the mechanical properties of BP-PENK polymer films are shown in Table 3. The first two columns show the tensile strength and elongation at the break of BP-PENK copolymers. The tensile strength and elongation at break gradually increased with the increase in the DFBP content. When the DFBP content reached 30%, the tensile strength and elongation at the break of the BP-PENK copolymer reached the maximum, which was 109.9 MPa and 45.2%. This was because the increase in the polyaryletherketone segment in the molecular backbone increased the strength of the copolymer molecular backbone, resulting in an increase in the tensile strength. At the same time, due to the increase in the polyaryletherketone segment, the copolymer changed from elastic deformation to yield, which improved the elongation at the break of the copolymer. The last column shows the modulus of elasticity of BP-PENK, which exceeded 2100 MPa for all the copolymers. Overall, BP-PENK30 had the best mechanical properties.

**Figure 5:**
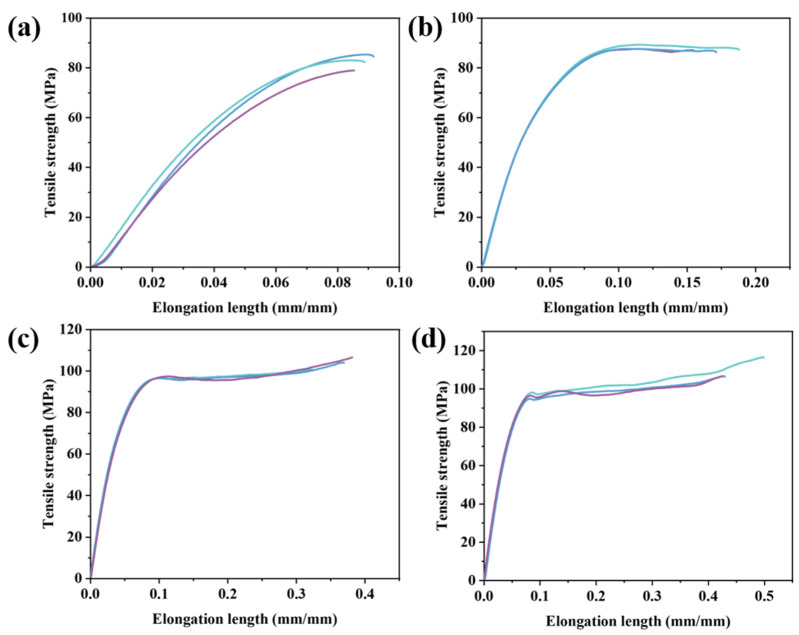
Stress–strain curves of BP-PENK polymers (**a**) PENK0, (**b**) PENK20, (**c**) PENK 25, (**d**) PENK30.

The stress–strain curves of the polymers are shown in Figure 5, and different colors in the same figure correspond to multiple mechanical test samples of the same resin. It can be seen that the distribution of the stress-strain curve is more concentrated, which better reflects the mechanical properties of the polymer. The stress–strain curve of biphenyl-type PEN, as shown in Figure 5a, belongs to the clear elastic deformation stage. With the increase in the chain segments of polyarylether ketone, the polyether ketonitrile polymer gradually changed from the elastic deformation stage to the yield stage. The mechanical properties of PENK30 were the best among the polymers.

### 3.4. Dielectric Properties of BP-PENK Polymers

**Figure 6:**
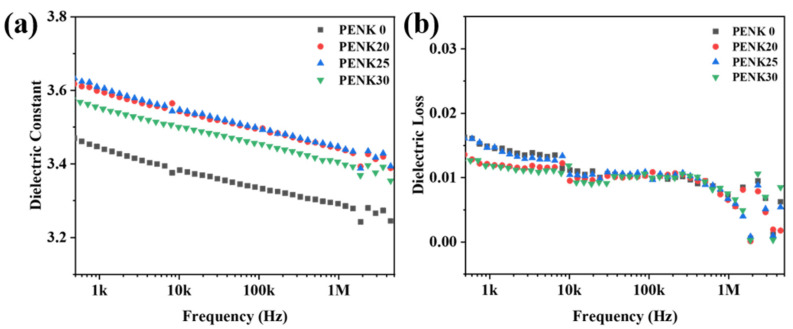
Dielectric properties of BP-PENK polymers (**a**) Dielectric constant of BP-PENK polymers, (**b**) Dielectric loss of BP-PENK polymers.

The dielectric data of the polymer films are shown in Figure 6. Figure 6a shows the dielectric constant of BP-PENK series films. Due to the frequency change, the polarization rate of the polar material cannot catch up with the frequency change, and there is a certain hysteresis phenomenon, which is shown on the graph as a gradual decrease in the dielectric constant with increasing frequency. The dielectric constant of pure PEN at 1 kHz is 3.45. As the number of polyaryletherketone chain segments in the polymer increases, the dielectric constant of the polymer is higher than that of pure PEN, which is due to the crystallization of the polymer, which leads to an increase in the density of the film. At the same time, the side chain cyano regular arrangement makes the polymer more polar, and the dielectric constant increases. The dielectric loss of the polymers is shown in Figure 6b, and the dielectric loss of BP-PENK at 1 kHz is lower than 0.015, which consumes less power. Therefore, the polymer has good dielectric properties.

### 3.5. Crystallization of the Thermally Treated BP-PENK Films

**Figure 7:**
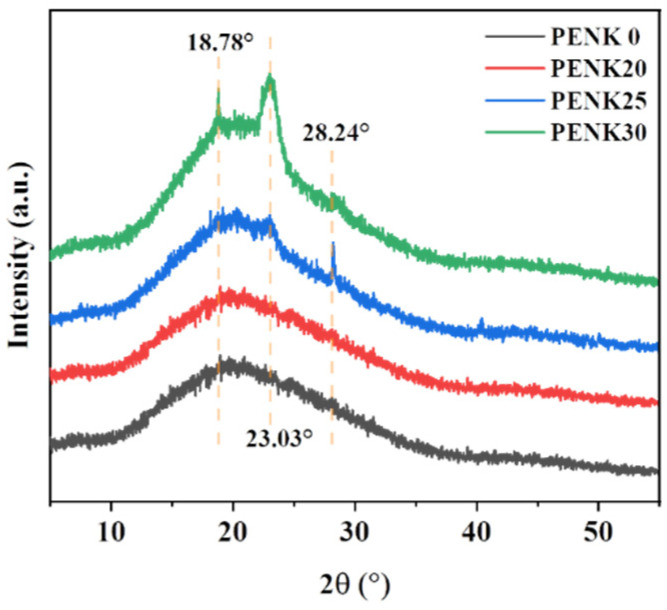
XRD curves of BP-PENK polymers.

In order to investigate the crystallization of BP-PENK films after high-temperature treatment, X-ray diffraction was used to test the crystallization of BP-PENK films. The XRD images of the heat-treated samples at 280 °C and a scanning angle of 5–55° are shown in Figure 7. With the increase in the polyaryletherketone chain segments, the area of the broad and large bun peaks of the polymer gradually decreases, and the area of the sharp and narrow strong diffraction peaks increases, which indicates that the crystallinity of the polymer increases gradually. Meanwhile, the diffraction peaks of the polymer appear at 18.78°, 23.03° and 28.24°, in which the diffraction peaks at 18.78° and 28.24° belong to the polyaryletherketone chain segments exclusively. It can be seen that with the increase in the polyaryletherketone chain segments, the intensity of the diffraction peaks of the polymer at two places also increases gradually, and the crystallinity of the polymer increases.

### 3.6. Thermal Properties of the Thermally Treated BP-PENK Films

**Figure 8:**
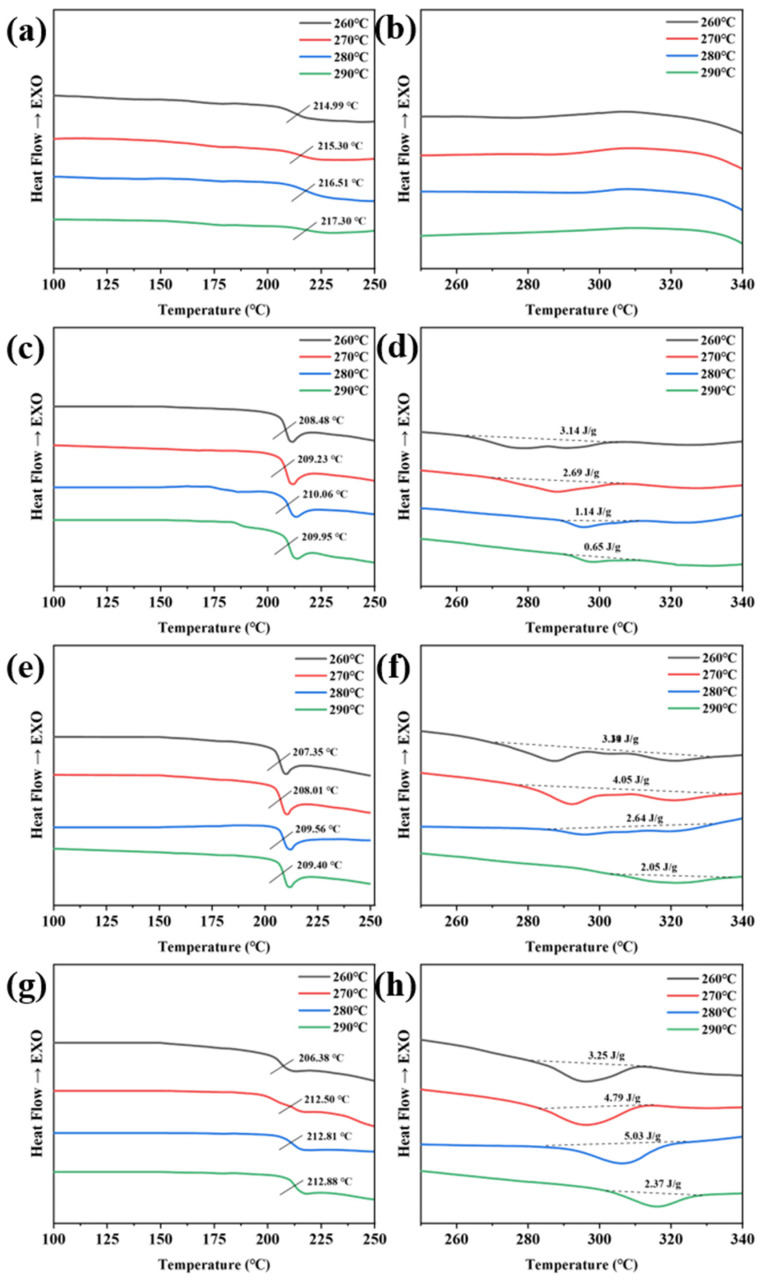
DSC curves of the thermally treated BP-PENK (**a**) Tg curve of PENK0, (**b**) Tm curve of PENK0, (**c**) Tg curve of PENK20, (**d**) Tm curve of PENK20, (**e**) Tg curve of PENK25, (**f**) Tm curve of PENK25, (**g**) Tg curve of PENK30, (**h**) Tm curve of PENK30.

The glass transition temperature (*T_g_*) and melting of BP-PENK after isothermal heat treatment were tested and analyzed via DSC. The DSC curves of the polymers treated at different temperatures for 3 h are shown in Figure 8 and the specific data are shown in Table 4. The glass transition temperature (*T_g_*) and melting temperature (*T_m_*) increased as the treatment temperature increased. This is because the glass transition temperature is the temperature at which the polymer molecular chains begin to move. Isothermal treatment promotes polymer crystallization, and the crystalline zone restricts the movement of the chain segments, resulting in an increase in *T_g_*. The DSC curves of PENK0 are shown in Figure 8a,b. Its glass transition temperature slightly increased, and no melting peaks were observed in the tested temperature range. The enthalpy of the melting of PENK20 decreased with the increase in processing temperature. This is due to the fact that the main chain of the molecule in the polyarylene ether nitrile chain segment accounts for the main part, while warmth destroys the original crystal structure, which is not conducive to its crystallization. The enthalpy melting of PENK25 increases with the increase in the treatment temperature, and the temperature increase is favorable for its crystallization. When the temperature exceeds 270 °C, the original crystal structure is destroyed, which is unfavorable for crystallization. The melting enthalpy of PENK30 increases with the increase in the treatment temperature and decreases when the temperature exceeds 280 °C. Among these four structures, the enthalpy melting of BP-PENK30 was the largest, and its optimal heat treatment temperature was 280 °C, at which the enthalpy of melting was 5.02 J/g. The enthalpies of melting for PENK20-280, PENK25-280, and PENK30-280 in the DSC curves were 1.14 J/g, 2.64 J/g, and 5.03 J/g, showing an increasing trend. This is consistent with the gradual increase in crystallinity in the XRD curves under heat treatment at 280 °C. In addition, the melting peak of the sample was bimodal [41,42,43,44] at a lower treatment temperature and gradually changed from bimodal to unimodal with the increase in the treatment temperature. It shows that with the increase in the treatment temperature, the crystals were rearranged, and the crystals gradually tended to be perfect, and the imperfect crystals disappeared. Therefore, the thermal properties of polymers can be improved by isothermal thermal treatment.

**Figure 9:**
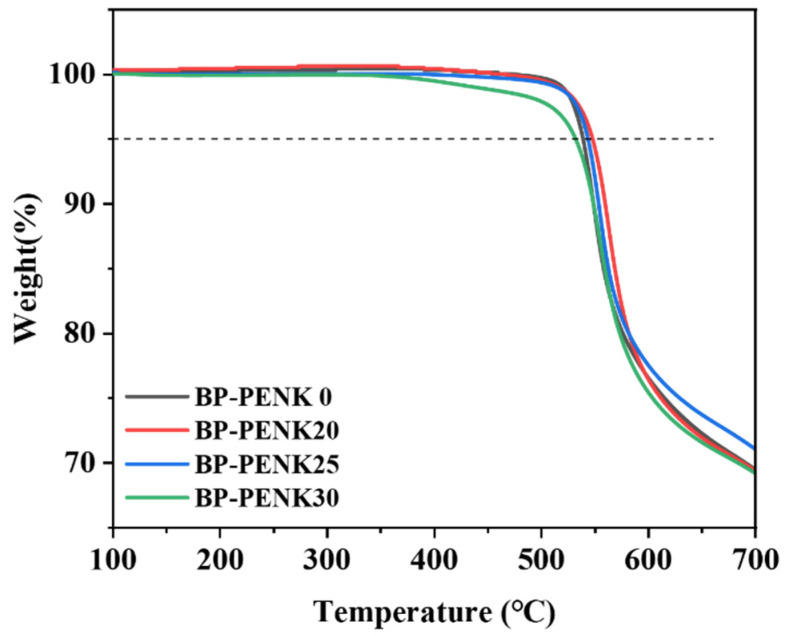
TGA curve of the thermally treated BP-PENK.

In addition, the thermal stability of BP-PENK after heat treatment at 280 °C was also tested using thermogravimetric analysis (TGA), and the TGA curves are shown in Figure 9. The 5% decomposition temperatures (*T_d_*_5%_) of the copolymers were all greater than 530 °C, and the copolymers had good thermal stability.

### 3.7. Dielectric Properties of the Thermally Treated BP-PENK Films

**Figure 10:**
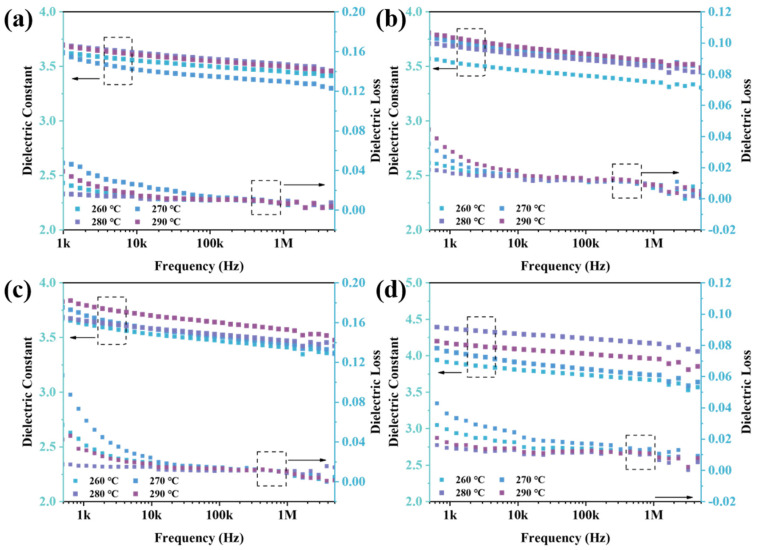
Dielectric properties of thermally treated BP-PENK films (**a**) PENK0, (**b**) PENK20, (**c**) PENK25, (**d**) PENK30.

The test data of the dielectric properties of isothermal heat-treated polymer films in the range of 500 Hz–5 MHz are shown in Figure 10. With the increase in frequency, the dielectric constant and dielectric loss of PEN copolymer films decreased due to polarization relaxation [45]. Among them, the dielectric constant of PENK0 films at different temperature treatments is shown in Figure 10a. The dielectric constants all increased, probably due to the more regular cyano arrangement of the side chains of the polymer molecules via heat, and the polarity of the polymers increased; thus, the dielectric constants increased. Figure 10b–d show the dielectric constants of PENK20, PENK25, and PENK30 at different treatment temperatures. The dielectric constants of the polymers all increased after isothermal heat treatment. On the one hand, this was due to an increase in the crystallinity of the polymer, which led to an increase in the density of the polymer and an increase in the dielectric constant. On the other hand, the isothermal treatment rearranged the polymer molecular chains so that the polar cyano groups were more regularly arranged on one side of the molecular chain, resulting in an increase in molecular polarity and a consequent increase in the dielectric constant. Among them, the most significant increase in the dielectric constant was observed for PENK30 after isothermal treatment, which is also consistent with the measured DSC results. When the treatment temperature was 280 °C, the dielectric constant of PENK30 increased from 3.55 to 4.39, which had the best dielectric properties. Therefore, PENK30 was subsequently selected for the breakdown test. The dielectric loss of the BP-PENK polymer is shown in Figure 10a–d, which increased after the heat treatment but still remained at a low level, showing good dielectric properties.

### 3.8. Crystallization Properties of the Thermally Treated BP-PENK Films

**Figure 11:**
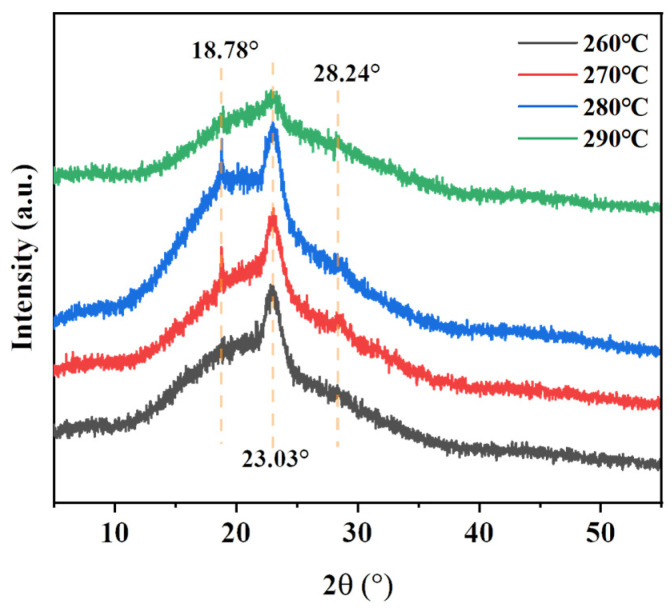
XRD curves of BP-PENK30 at different temperatures.

In order to study the crystallization of PENK30 after high-temperature treatment, X-ray diffraction was used to test the crystallization of the films [46]. The XRD images obtained are shown in Figure 11. The diffraction peaks of the polymer were located at 18.78°, 23.03°, and 28.24°, respectively. Different temperature treatments did not change the positions of the diffraction peaks, indicating that the elevated temperature did not change the crystalline shape of the structure.

In addition, the diffraction peaks of BP-PENK30 at different temperatures were fitted using Jade 6 software. The crystallinity of the polymers was calculated using Equation (2), and specific data are shown in Table 5.

(2) $$x_{c}=\frac{S_{c}}{S_{c}+S_{a}}$$

In Equation (2), $x_{c}/\%$ is the crystallinity and $S_{c}/m^{2}$ is the area of the strong diffraction peak in the XRD curve, which is positively correlated with the crystal volume; $S_{a}/m^{2}$ is the area of the bun peak in the XRD curve, which is positively correlated with the amorphous volume. It can be seen that the crystallinity of BP-PENK30 increased from 7.00% to 11.83% with an increasing treatment temperature and decreased to 3.49% when the temperature exceeded 280 °C, indicating that too high a temperature is not conducive to crystallization. Meanwhile, the enthalpies of melting for PENK30-260, PENK30-270, PENK30-280, PENK30-290 were known from the previous DSC curves to be 3.25 J/g, 4.79 J/g, 5.03 J/g, and 2.37 J/g, respectively. And the crystallization of PENK30-260, PENK30-270, PENK30-280, and PENK30-290 was 7.00%, 10.49%, 11.83%, 3.49% obtained using Jade 6 software fitting, which is in line with the trend of the DSC enthalpy of melting, which firstly increased and then decreased.

### 3.9. Energy Storage Properties of the Thermally Treated BP-PENK Films

The energy storage performance of dielectric materials is an important indicator for assessing their application value, which is related to the basic physical properties of the dielectric. High energy storage density can significantly reduce the volume and weight of capacitors and accessories. The energy storage density of film capacitors can be expressed as Equation (3).

(3) $$U=\frac{1}{2}\varepsilon_{0}\varepsilon_{r}E_{b}^{2}$$

**Figure 12:**
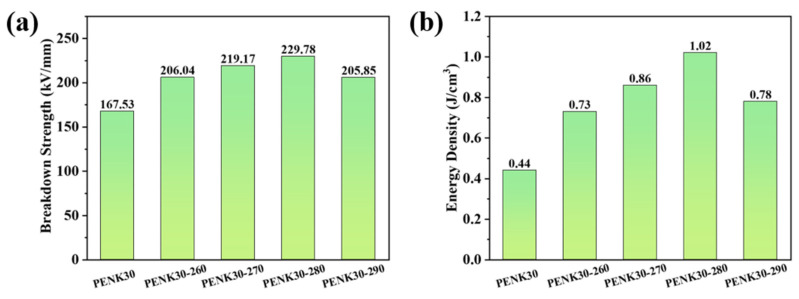
(**a**) Breakdown strength of PENK30, (**b**) Energy density of PENK30.

In Equation (3), $\varepsilon_{0}\;$is the vacuum dielectric constant,$\;\varepsilon_{0}=8.854\times 10^{-12}\;F/m$, $\varepsilon_{r}$ is the relative dielectric constant, and $E_{b}$ is the breakdown strength of the material. Figure 12a shows the breakdown strength of the copolymer PENK30 after different temperature treatments. The breakdown strength of PENK30 was 167.53 kV/mm, and the breakdown strength of the polymers was all enhanced after high-temperature treatment. Among them, the film treated at 280 °C had the best breakdown strength of 229.78 kV/mm, which was 37.16% higher compared to the untreated PENK30 film. This was due to the fact that after high-temperature treatment, the crystallinity of the polymer film increased, and the arrangement was more regular, while the inner part of the material was denser, which was not easy to puncture. The energy storage density calculated from Equation (3) is shown in Figure 12b. The energy storage density of the untreated PENK30 film was only 0.44 J/cm^3^, but the dielectric constant, as well as the breakdown strength of the film, increased after high-temperature treatment. Since the energy storage density was positively correlated with the dielectric constant and the square of the breakdown strength, there was a significant increase in the energy storage density of the films after high-temperature treatment. The energy storage density of the films treated at 280 °C was the largest, reaching 1.02 J/cm^3^, which is 131.82% higher than that of the untreated films. This indicates that the method of isothermal thermal treatment is a good method to enhance the energy storage density of the material.

## 4. Conclusions

In this paper, BP-PENK copolymers containing different proportions of DCBN and DFBP (BP-PENK0, BP-PENK20, BP-PENK25, BP-PENK30) were successfully prepared via condensation polymerization. The properties of BP-PENK polymers were tested using infrared spectroscopy (FTIR), DSC, and TGA. After the isothermal thermal treatment of the BP-PENK film, various properties and the crystallization of the BP-PENK film at 260 °C, 270 °C, 280 °C, and 290 °C were studied. The mechanical test showed that the mechanical properties of the copolymer gradually improved with the increase in the DFBP content. BP-PENK30 has the best mechanical properties, with a tensile strength of 109.9 MPa and an elongation at a break of 45.2%. After being treated at 280 °C for 3 h, the melting point of BP-PENK30 was 306.71 °C, and the melting enthalpy was 5.02 J/g. Its crystallinity reached 11.83% via XRD fitting. In addition, its energy density reached 1.02 J/cm^3^, which was enhanced by 131.82% compared with PENK30. The results show that the crystalline properties of the copolymers can be improved by isothermal thermal treatment, which, in turn, enhances the energy storage density of the materials.

## Figures and Tables

**Table 1:** Composition of BP-PENK copolymers.

Polymer	DCBN (mol)	DFBP (mol)	BP (mol)	K_2_CO_3_ (mol)	NMP (mL)	Toluene (mL)
PENK0	0.1500	0.0000	0.1500	0.2100	81	27
PENK20	0.1200	0.0300	0.1500	0.2100	81	27
PENK25	0.1125	0.0375	0.1500	0.2100	81	27
PENK30	0.1050	0.0450	0.1500	0.2100	81	27

**Table 2:** Detailed thermal data of BP-PENK polymer.

Sample	PENK0	PENK20	PENK25	PENK30
*T_g_* (°C)	213.27	207.99	202.25	200.76
*T_g_*′ (°C)	213.54	207.90	202.45	200.60
*T_g_*″ (°C)	213.88	206.59	202.59	200.95
*T_d_*_5%_ (°C)	550.07	555.68	533.38	542.13

**Table 3:** Detailed data of the mechanical properties of BP-PENK.

Sample	Tensile Strength (MPa)	Elongation at Break (%)	Elasticity Modulus (MPa)
PENK0	82.4 ± 2.6	8.9 ± 0.3	1770.3 ± 20.8
PENK20	88.2 ± 1.0	17.2 ± 1.6	2172.3 ± 35.0
PENK25	103.7 ± 2.9	35.9 ± 3.0	2291.2 ± 73.0
PENK30	109.9 ± 5.8	45.2 ± 4.1	2140.1 ± 95.0

**Table 4:** Detailed thermal data for BP-PENK.

Sample	*T_g_* (°C)	*T_m_* (°C)	$\mathrm{Melting}\;\mathrm{Enthalpy}\;\Delta H_{f}$ (J/g)
PENK0-260	214.99	-	-
PENK0-270	215.30	-	-
PENK0-280	216.51	-	-
PENK0-290	217.30	-	-
PENK20-260	208.48	278.08	3.14
PENK20-270	209.23	287.96	2.69
PENK20-280	210.06	295.66	1.14
PENK20-290	209.95	297.85	0.65
PENK25-260	207.35	287.12	3.39
PENK25-270	208.01	292.18	4.05
PENK25-280	209.56	296.91	2.64
PENK25-290	209.40	318.61	2.05
PENK30-260	206.38	295.78	3.25
PENK30-270	212.50	296.22	4.79
PENK30-280	212.81	306.71	5.03
PENK30-290	212.51	315.55	2.37

**Table 5:** Crystallinity of PENK30.

Heat Treatment Temperature	Crystallinity
260 °C	7.00
270 °C	10.49
280 °C	11.83
290 °C	3.49

## Data Availability

Data are contained within the article.
